# To Contributors

**Published:** 1859-06

**Authors:** 


### TO CONTRIBUTORS.

Our hearts are overflowing to the many kind friends who have of late remembered the original department of our journal. They have, indeed, been so liberal that we find ourselves compelled to apologize for the non-appearance of articles that have been on hand a considerable length of time. We have had some hesitation in making this statement, for fear that it might be misconstrued, and that there might arise the impression, that no necessity existed for farther contributions. We would however say, that we have never been more anxious to receive original articles, and hope that our friends will not relax their exertions in this particular. Unless a considerable number of original contributions are constantly on hand, it is impossible that a journal can be properly sustained. Indeed, we would greatly prefer to publish an original journal, save in a passing glance at medical improvement going on throughout the world, if we could command the material which is *laid up* in the mind and experience of the medical men of the south.

We would then say to all those who feel an interest in medical science, and especially to those who feel an interest in this journal, as an humble co-laborer in this great work, we are ever thankful for any aid furnished, and if their contributions do not appear in its pages as soon as they may expect, they may at all times rest assured that it is not because they are not appreciated.

